# Supplementary material for: Full-Length OmpA: Structure, Function, and Membrane Interactions Predicted by Molecular Dynamics Simulations
Source: Biophys J. 2016 Oct 18;111(8):1692–702. doi: 10.1016/j.bpj.2016.09.009 (PMC5071624; doi:10.1016/j.bpj.2016.09.009)
Supplement: Document S1. Figs. S1–S3 [file mmc1.pdf]

Biophysical Journal, Volume *111*

Supplemental Information

Full-Length OmpA: Structure, Function, and Membrane Interactions Predicted by Molecular Dynamics Simulations

Maite L. Ortiz-Suarez, Firdaus Samsudin, Thomas J. Piggot, Peter J. Bond, and Syma Khalid

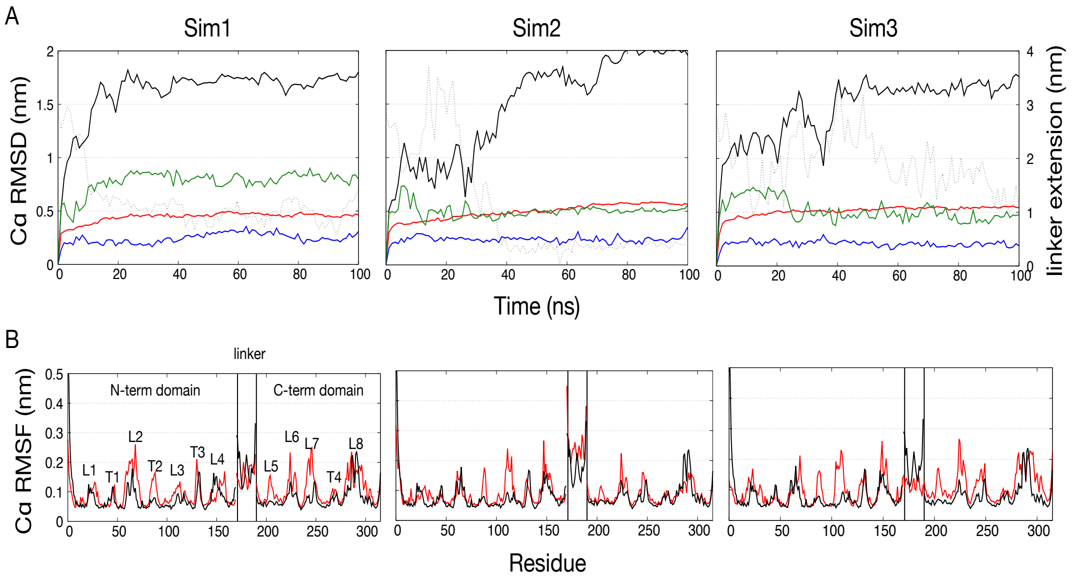

Supp. Figure 1: OmpA monomer structural stability. (A) Cα RMSD of the full length OmpA monomer (black) and its domains, i.e. N-terminal domain (red), linker (green) and C-terminal domain (blue). The extension of the linker region is depicted as black dashed line. (B) Per-residue Cα RMSF of OmpA monomer from the isolated monomeric simulation (red), compared to that from dimeric simulation (black). Each domain is separated by the black vertical lines.

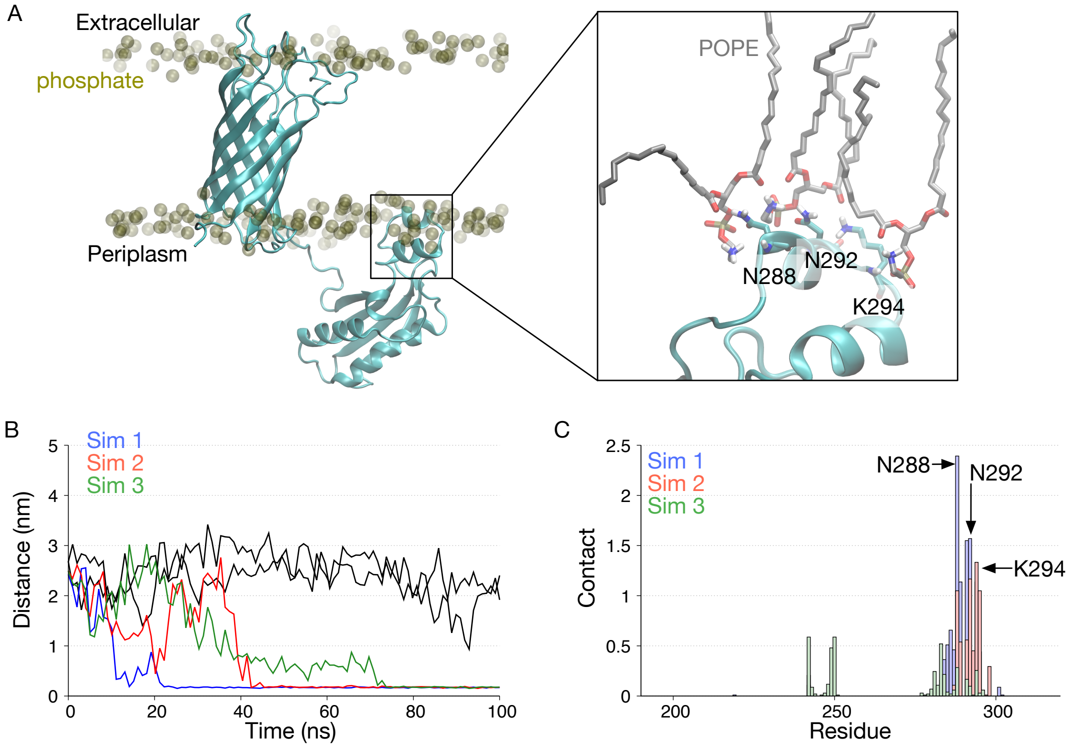

Supp. Figure 2: OmpA C-terminal domain interacts with the membrane. (A) Representative structure of OmpA monomer with respect to the membrane (phosphate atoms shown as brown spheres) at the end of the 100 ns simulations. Enlarged figure highlights key residues involved in the interactions. (B) Minimum distance between the C-terminal domain (residue 188-316) and the inner leaflet of the outer membrane throughout the 100 ns simulations (blue, red and green), compared to that of the dimer simulations (black). (C) Contact analysis performed for each residue of the C-terminal domain, presented as the average number of lipids found within 3.5 Å of the residue.

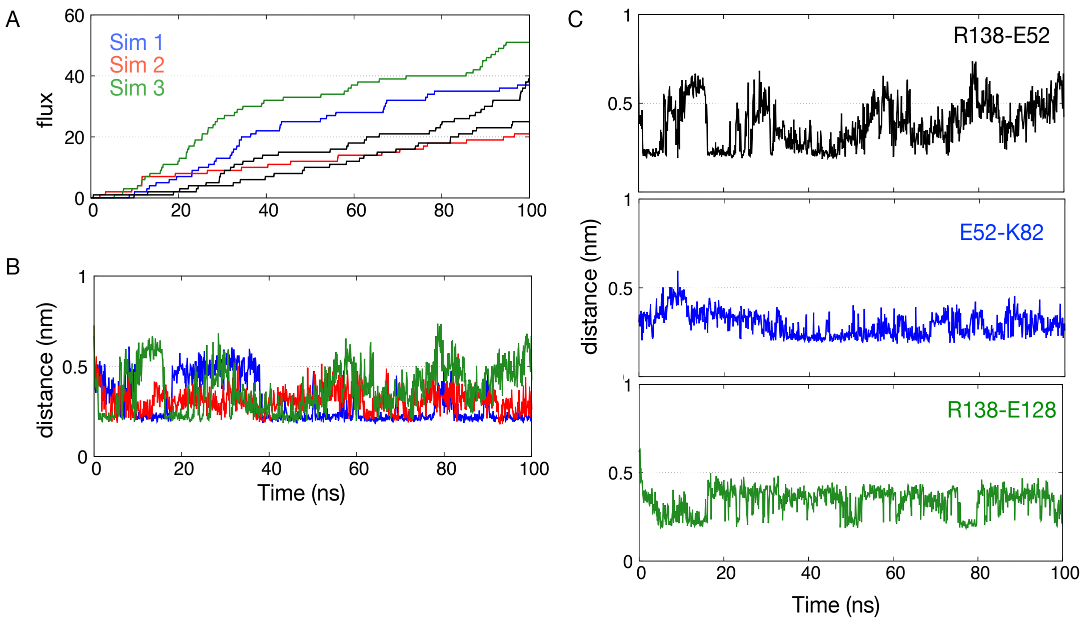

Supp. Figure 3: Water transport through the monomeric OmpA. (A) Total flux of water molecules through the N-terminal β-barrel throughout the 100 ns simulation of OmpA monomer (blue, red and green lines), compared to that of OmpA dimer (black lines). (B) The corresponding opening and closing of the R138-E52 gate depicted as the minimum distance between these residues. (C) The interactions between these pore gating residues with two other key residues in Simulation 3, shown as minimum distance between E52 and K82 (blue), and R138 and E128 (green).
