# Supplementary material for: Full-Length OmpA: Structure, Function, and Membrane Interactions Predicted by Molecular Dynamics Simulations
Source: Biophys J. 2016 Oct 18;111(8):1692–702. doi: 10.1016/j.bpj.2016.09.009 (PMC5071624; doi:10.1016/j.bpj.2016.09.009)
Supplement: Document S2. Article plus Supporting Material [file mmc2.pdf]

Biophysical Journal
Article

Biophysical Society

Full-Length OmpA: Structure, Function, and Membrane Interactions Predicted by Molecular Dynamics Simulations

Maite L. Ortiz-Suarez,[1] Firdaus Samsudin,[1] Thomas J. Piggot,[1] Peter J. Bond,[2,3,*] and Syma Khalid[1,*]
[1]School of Chemistry, Highfield Campus, University of Southampton, Southampton, United Kingdom; [2]Bioinformatics Institute (A*STAR), Singapore, Singapore; and [3]Department of Biological Sciences, National University of Singapore, Singapore, Singapore

ABSTRACT OmpA is a multidomain protein found in the outer membranes of most Gram-negative bacteria. Despite a wealth of reported structural and biophysical studies, the structure-function relationships of this protein remain unclear. For example, it is still debated whether it functions as a pore, and the precise molecular role it plays in attachment to the peptidoglycan of the periplasm is unknown. The absence of a consensus view is partly due to the lack of a complete structure of the full-length protein. To address this issue, we performed molecular-dynamics simulations of the full-length model of the OmpA dimer proposed by Robinson and co-workers. The N-terminal domains were embedded in an asymmetric model of the outer membrane, with lipopolysaccharide molecules in the outer leaflet and phospholipids in the inner leaflet. Our results reveal a large dimerization interface within the membrane environment, ensuring that the dimer is stable over the course of the simulations. The linker is flexible, expanding and contracting to pull the globular C-terminal domain up toward the membrane or push it down toward the periplasm, suggesting a possible mechanism for providing mechanical stability to the cell. The external loops were more stabilized than was observed in previous studies due to the extensive dimerization interface and presence of lipopolysaccharide molecules in our outer-membrane model, which may have functional consequences in terms of OmpA adhesion to host cells. In addition, the pore-gating behavior of the protein was modulated compared with previous observations, suggesting a possible role for dimerization in channel regulation.

INTRODUCTION

One of the most abundant outer membrane proteins (OMPs) in *Escherichia coli* is OmpA, with typically 100,000 copies per cell. The N-terminal domain (NTD) of OmpA is an eight-stranded β-barrel that resides within the OM. A 15-amino-acid linker region connects the barrel to the soluble C-terminal domain (CTD). The CTD is located in the periplasm, a gel-like region that separates the OM from the inner cytoplasmic membrane in Gram-negative bacteria, of which *E. coli* is an archetypal example.

The structure of the β-barrel of OmpA has been determined with the use of x-ray and NMR techniques, and has been the focus of a number of biophysical and simulation studies (1–8). Although the structure of the CTD of OmpA from *E. coli* has not been determined, several structures of homologs from other species have been reported (9,10). The structure of the linker region is also not known, although it is thought that it is likely unstructured. Both the CTD and the linker domain have been largely neglected in biophysical and simulation studies. Recently, an experimentally validated static model of the full-length OmpA dimer was proposed by Robinson and co-workers (11). Mass-spectrometry data showed conclusively that full-length OmpA can form dimers that are mediated by interactions between the CTDs. Indeed, the authors suggested that full-length OmpA exists in equilibrium between the monomeric and dimeric states. Additional supporting evidence for the model was subsequently provided via low-resolution, small-angle x-ray scattering (SAXS) studies (12). Until now, however, the molecular details of the conformational dynamics and membrane interactions of the full-length protein have remained elusive. We note that the functional relevance of the OmpA dimer is also not known, but the evidence for its existence is compelling.

OmpA serves a number of functions in *E. coli* (13). It appears to be critical for adherence to plant surfaces in

Submitted June 7, 2016, and accepted for publication September 6, 2016.

*Correspondence: s.khalid@soton.ac.uk or peterjb@bii.a-star.edu.sg

Maite L. Ortiz-Suarez and Firdaus Samsudin contributed equally to this work.

Editor: Bert de Groot.
http://dx.doi.org/10.1016/j.bpj.2016.09.009

© 2016 Biophysical Society.
This is an open access article under the CC BY license (http://creativecommons.org/licenses/by/4.0/).

CrossMark

the enterohemorrhagic O157:H7 strain, and for binding to epithelial cells by meningitic *E. coli* (14). Its expression has also been associated with serum resistance in a neonatal rat model (15). OmpA has been proposed to function as a porin in *E. coli* (8). The channel-gating mechanism has been suggested to involve the disruption of a central salt bridge, E52–R138, by the nearby residues K82 and D128 in the NTD (1,3,8). Models involving this salt-bridge rearrangement yielded a predicted membrane conductance similar to those observed experimentally. Larger conductances were seen only in experimental studies of full-length OmpA, and the N-terminal barrel showed small conductance behavior, suggesting that the CTD may contribute to the formation of larger pores in the transmembrane barrel (11).

Another key functional aspect of OmpA is that the globular CTD interacts noncovalently with the bacterial cell wall and hence links the periplasm to the OM in which the NTD barrel resides, thereby providing mechanical strength to the cell. However, the localization and arrangement of the CTD relative to the NTD barrel within the periplasm remain poorly characterized.

The Gram-negative bacterial cell envelope comprises two lipid membranes. The inner membrane is a phospholipid bilayer composed of a mixture of lipids, with essentially the same composition for both leaflets. In contrast, the OM is an asymmetric bilayer: the inner leaflet is composed of a mixture of phospholipids, whereas the outer leaflet is composed almost completely of lipopolysaccharide (LPS). The cell wall is made of long glycan chains cross-linked by flexible peptide bridges, forming an elastic matrix known as peptidoglycan, where the globular CTD of OmpA resides. Thus, the environment in which OmpA is found in vivo is inherently complex; however, to date, no simulation studies and only a few experimental studies have addressed the molecular complexities of the membrane composition and oligomerization of OmpA. In summary, although the membrane-protein biophysics community has made tremendous progress in unraveling the myriad functions of OmpA, currently neither the conformational dynamics of the full-length protein, the functional relevance of the dimeric state, nor the interactions of the individual domains with the *E. coli* cell envelope are understood at the molecular level. Such an understanding is of fundamental importance for elucidating the precise structure-function relationships of this protein and consequently its impact of the integrity of the *E. coli* cell envelope.

To address this issue, we conducted the first, to our knowledge, atomistic-level molecular-dynamics simulation study of monomeric and dimeric, full-length OmpA in an LPS-containing model of the OM. We explored the dynamical behavior of the full-length OmpA structure reported by Marcoux et al. (11). Our simulations show that the conformations of the model in both the monomeric and dimeric states are stable in a realistic membrane environment, with conformational stability comparable to that observed in simulations of other LPS-embedded OMPs (1–4). The dimer maintains a well-conserved dimerization interface with a total buried surface area (BSA) of ~45 nm^2 among the three domains. We observed that the belts of aromatic residues on the surface of the β-barrels stabilized the dimerization and membrane/solvent interfaces. Our simulations provide a link between biophysical and structural data by showing that the linker is flexible and may aid in adapting to changes in the cell envelope, helping to maintain mechanical support. Further, they show that the external loops are stabilized by dimerization and interaction with LPS molecules, and thus may play a role in modulating adherence to host sites.

MATERIALS AND METHODS

Simulation systems

Full-length OmpA monomeric and dimeric models were provided by Marcoux et al. (11). The model presented here was constructed from the x-ray structure of the *E. coli* NTDs (PDB: 1G90), an 18-residue linker from the OmpA NMR structure of Klebsiella *pneumoniae* (PDB: 2K0L), and a homology model of the CTD based on Salmonella *enterica* (PDB: 4ERH; 94% sequence identity), spanning residues 1–316. The outer leaflet of the EcOM bilayer was composed entirely of Ra LPS molecules, which consist of lipid A and the full core of the LPS (16). The full core used for the Ra LPS was of the R1 core type, which is the most common core composition in *E. coli* (17,18). The inner leaflet of the membrane was composed of a mixture of phosphatidylethanolamine (PE; 90%), phosphatidylglycerol (PG; 5%), and diphosphatidylglycerol (DPG; 5%) phospholipids (19). The phospholipid fatty acyl tail of the inner leaflet was composed of 1-palmitoly, 2-cis-vaccenyl (PV) for PE and PG and 1-palmitoyl, 2-cis-vaccenyl, 3-pamitoly, 4-cis-vaccenyl (PVPV) for DPG. The force-field parameters for LPS and phospholipids were as described and validated previously (20).

Simulation protocols

All simulations were performed using the GROMACS package (version 4.6.1), the GROMOS 54A7 force field, and a simple-point-charge water model (21–24). After membrane embedding and solvation, the systems were equilibrated using a restrained NVT run of 100 ns. A 10 ns NPT equilibration was run to allow the lipid tails to close any gaps in the bilayer, restraining the protein and lipid headgroups only. During the simulations, the LPS, phospholipids, and solvent (water plus ions) were maintained at a constant temperature above the membrane gel (L_β) to liquid crystal (L_α) phase transition temperature (313 K) via a Nosé-Hoover thermostat with a time constant of 0.5 ps (25–27), as described previously (28). A pressure of 1 bar was maintained by using semi-isotropic pressure coupling with a Parrinello-Rahman barostat and a time constant of 5 ps (29). Electrostatic interactions were treated using the smooth particle mesh Ewald algorithm with a short-range cutoff of 0.9 nm (30). van der Waals interactions were truncated at 1.4 nm with a long-range dispersion correction applied to the energy and pressure. The neighbor list was updated every five steps during the simulations. All bonds were constrained using the LINCS algorithm, allowing a 2 fs time step to be applied (31). These simulation parameters were chosen to replicate those used in the work of Kukol (32), on which the phospholipid parameters used in these simulations were originally based. Two different solvation protocols were used. In low-ionic-strength simulations, sufficient counterions were carefully added to neutralize the system charge and avoid long-range electrostatic artifacts due to the highly charged LPS leaflet. In these simulations, the neutralizing salt concentration was equivalent to ~0.3 M. In high-ionic-strength simulations, Mg^{2+} and Cl^- ions

were added to a concentration of ~1 M instead. We note here that Mg^{2+} ions are located primarily at the LPS headgroups and are essential for maintaining the integrity of the bilayer. Thus, the salt concentration in the bulk water region is much lower, similar to conditions in vivo (5).

RESULTS

The full-length OmpA monomer and dimer were embedded in a complex OM model containing LPS in the outer leaflet and a combination of the phospholipids PE, PG, and DPG in the periplasmic leaflet, surrounded by a water environment with neutralizing counterions equivalent to ~0.3 M. Since Carpenter et al. (33) previously reported an alteration in the dynamics of the OmpA barrel of *Pasteurella multocida* upon a change in the ionic strength of the simulation system, we also performed simulations of the dimer in an ~1 M $MgCl_2$ solution. Simulations of the monomer were performed for 100 ns, with three independent repeats, whereas the dimers were simulated for 500 ns, with two independent repeats of each system. The results are summarized in Table 1.

Structural drift and stability of the secondary domains

Given that the simulations were initiated from a static model of the full-length protein, in our initial analyses of the simulations we sought to assess and quantify any conformational deviations from the model. We evaluated the structural drifts of the NTD (residues 1–172), the linker (residues 173–187), and the CTD (residues 188–316) by measuring their root mean-square deviations (RMSDs) from the starting model, as shown in Figs. 1 and S1 in the Supporting Material.

The overall drift of the Cα backbone atoms of the full protein reached a maximum value of <2 nm in both the monomer and dimer simulations. The Cα RMSDs were also calculated for each individual domain and these remained below 0.5 nm, with the N-terminal β-barrel deviating the least (~0.2 nm). For the full NTD, including loops and turns, the maximum deviation from our simulations was ~0.4 nm, which is ~6× higher than the final Cα RMSD reported from simulations of a single OmpA NTD in a phospholipid

TABLE 1 Summary of Simulations

Simulation	Ionic Strength	Size (nm^3)	Simulation Time (ns)	β-Barrel Cα RMSD (nm)[a]
OmpA Monomer	low	10.1 × 10.8 × 16.3	3 × 100	0.180
				0.196
				0.204
OmpA Dimer	low	”	2 × 500	0.211
				0.217
OmpA^{1M} Dimer	1 M	”	2 × 500	0.188
				0.173

[a]The values of the β-barrel Cα RMSDs relative to the starting model are the plateau values averaged across both proteins.

bilayer (1). The larger deviation in these simulations most likely reflects the fact that our simulations were up to 250× longer than those reported by Bond et al. (1) in 2002, and thus explored a greater region of the conformational landscape. However, it is worth noting that this deviation is only twice as high as that observed in another 10 ns simulation of monomeric OmpA performed in a 1,2-dimyristoyl-*sn*-glycero-3-phosphocholine (DMPC) bilayer, and is similar to that found in a simulation conducted in a micelle system (6).

Since a disordered linker region connects the NTD and CTD, its length over the course of the simulations was measured as the distance between the center of mass of the bottom of the N-terminal barrel and the start of the CTD (see Fig. 1 *C*). The extension of the linker varied throughout the simulations, starting at a length of ~2–3 nm, decreasing in length at 200–300 ns and ~50–250 ns in the first and second independent simulations, respectively, and then fluctuating to settle on a final length of ~3.3 nm in both simulations. This was a 0.5 nm longer plateau value than in the simulations of this system containing only neutralizing counterions (Fig. 1 *A*).

Intriguingly, for the isolated monomer state, the linker region contracted and reached a final length of ~0.5 nm. This was caused by interactions between the C-terminal periplasmic domain and the inner leaflet of the OM (Fig. S2). We found that in all three independent repeats, the interactions were mediated by residues 280–300. It is thus possible that these residues represent a conserved membrane-binding surface, although this awaits further rigorous confirmation. We note that such interactions were not observed in most of the simulations of OmpA dimer, most likely due to the close proximity of these residues to the dimerization interface, which would reduce their accessibility. OmpA dimerization, therefore, could serve as a mechanism to prevent contraction of this linker region and thus interaction with the OM, which may have biological significance with regard to cell size and mechanical strength.

Both experimental and simulation studies have shown that the loop regions that connect the strands of bacterial OM β-barrel proteins on the extracellular side are highly flexible. To investigate this in our simulations, we calculated the time-averaged root mean-square fluctuations (RMSFs) of the Cα atoms fitted to the loops and the turns of the NTD of OmpA. Interestingly, in general, both loops and turns revealed similar degrees of fluctuation, <0.2 nm. This is rather different from the findings reported by Bond et al. (1) for a single β-barrel domain in a DMPC bilayer, which showed much higher fluctuations in the loops compared with the turns. Notably, more flexible extracellular loops compared with periplasmic turns, with a few exceptions, were predicted from x-ray and NMR structures of other eight-stranded β-barrel proteins, such as PagP, NspA, and the 12-stranded OMPLA (34–37). This also held true for simulation studies of simple membranes and micelles

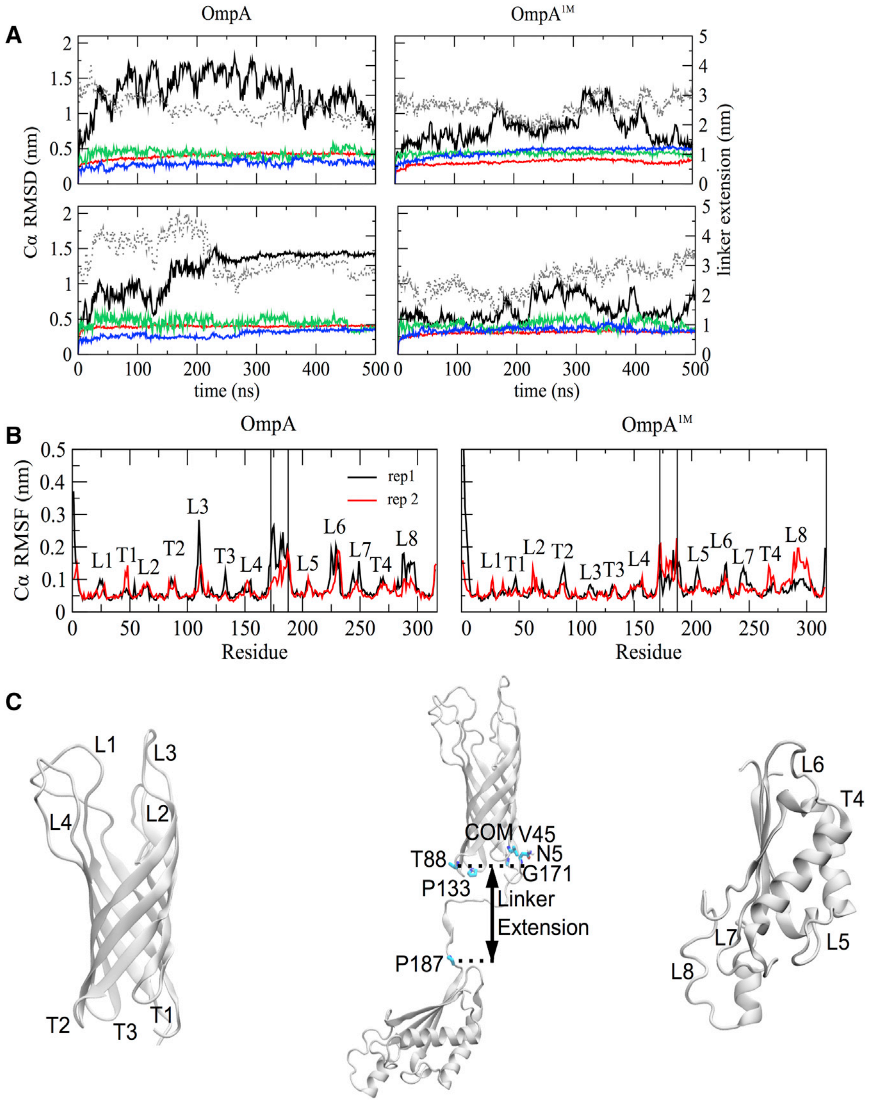

FIGURE 1 OmpA structural stability. (*A*) Cα RMSD of the overall protein and separate domains at low and 1 M ionic strengths compared with the extension of the linker region, as labeled in the inset. Results are shown for the least stable chain in each simulation replica. The different curves show data for the full protein (*black*), linker extension (*black dashed*), NTD (*red*), linker (*green*), and CTD (*blue*). A similar analysis for the monomer simulation is provided in Fig. S1. (*B*) Per-residue Cα RMSF of OmpA. For clarity, only the least stable chain is shown. Vertical lines represent domain boundaries. Fitting was done for each individual domain. (*C*) The structure of a full-length OmpA chain from *E. coli* is shown in cartoon format, showcasing three distinct domains: the transmembrane eight-stranded β-barrel NTD (*left*), a disordered linker, and a peptidoglycan-binding CTD (*right*). The linker extension was calculated as the center-of-mass distance between the periplasmic side of the NTD barrel, comprised of residues N5, V45, T88, P133, and G171, and the start of the CTD, namely, residue P187 (*center*). To see this figure in color, go online.

of the same proteins (38–40). The comparable flexibility of the turns and loops in our simulations is likely a consequence of both stabilization by the dimerization interface (particularly loops L1, L2, and L4) and the more sophisticated OM model we used, since the interactions with LPS led to reduced flexibility of the loops. The former possibility is supported by the slightly higher RMSF values recorded for the loops in the monomer simulations. The stabilization effect of the OM is in agreement with a previous simulation study of FecA, a TonB-dependent transporter of *E. coli*, in which the LPS molecules decreased the overall mobility and conformational sampling of the extracellular loops (2). We note that loop L3 exhibits greater fluctuation in low-salt conditions than in 1 M salt. This loop is farthest away from the dimerization interface and contains two lysine residues, which are able to interact with the phosphate groups of LPS. The greater RMSFs for L3 are largely due to just one simulation in low salt, in which the loop was observed to alternate between extending toward the membrane and being in a more compact conformation.

Conformational dynamics

For multidomain proteins, it is of interest to probe the nature of the local and global conformational rearrangements to gain insights into the structure-function relationships. For this purpose, we analyzed the simulations of dimeric OmpA via cluster analysis using the algorithm of Daura et al. (47). Each sampled conformation was assigned to a cluster based on an RMSD cutoff of 0.3 nm. The sizes of the clusters and a representative structure for the largest cluster are shown in Fig. 2.

Two independent simulations of OmpA dimer in low-salt concentrations showed different dynamics: the first simulation revealed a large conformational flexibility, as exemplified by a large number of clusters, whereas the dimer in the

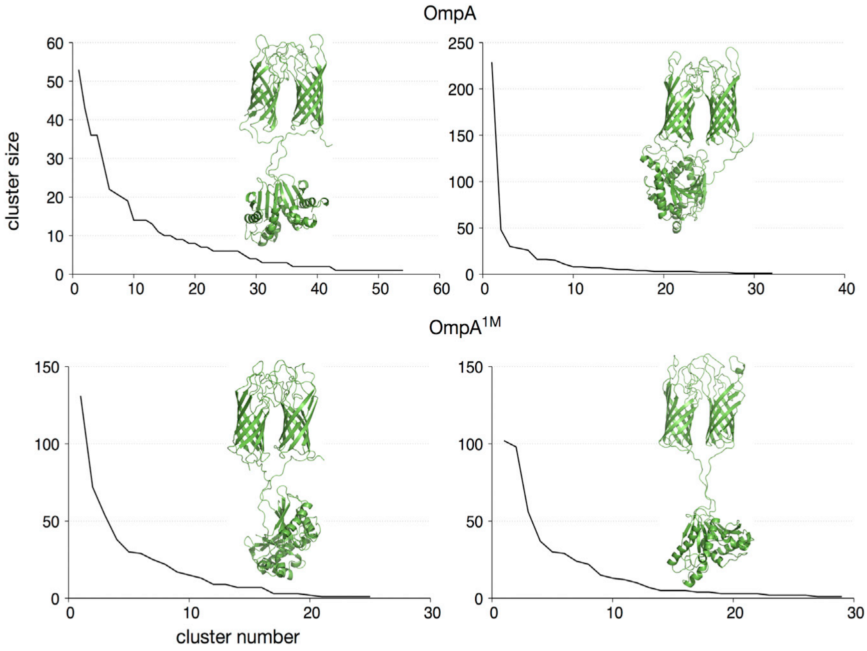

FIGURE 2 Cluster analysis for the OmpA dimer. The frequency of each cluster is shown, ordered from the most to least frequently sampled conformation. A representative structure of the most dominant cluster in each simulation is shown in the inset. To see this figure in color, go online.

second simulation sampled a much smaller conformational landscape, with around half of the structures belonging to the same cluster. Further inspection revealed that in the latter simulation, the C-terminal periplasmic domain of the protein interacted with the NTD after ~250 ns and remained in that conformation for the rest of the simulation. Such an interaction was not observed in the first simulation, and the dimer therefore has more conformational freedom. It is worth reiterating that the linker was flexible, alternating between extended conformations of up to 4.5 nm and contracted ones of only 2.0 nm in length, and thereby contributed to the different behaviors of the attached CTD observed in these two simulations.

Both simulations of the higher-ionic-strength system, on the other hand, displayed similar conformational dynamics (as indicated by their cluster sizes and representative structures), with the CTD remaining in the region equivalent to the periplasmic space throughout most of the simulations. In this case, the higher salt concentration helped to screen the C-terminal polar residues from the charged ethylamine and phosphate groups of the periplasmic lipids. The full-length OmpA monomer from *P. multocida* showed comparable motions. The CTD moved relative to the NTD and closer to the inner leaflet of the bilayer at low ionic concentrations, whereas at 1 M concentrations the major motion was a twisting of the CTD.

OmpA dimerization interfaces

Localization of the OmpA dimer in a complex model membrane resulted in an ~2.6-fold increase of the total BSA within the dimerization interface compared with the starting model, irrespective of the ionization strength (Table 2), suggesting the formation of a stable, lipid-solvated dimer.

To determine the BSA, we first used the maximal-speed molecular surfaces algorithm within VMD to calculate the solvent-accessible surface area (SASA). We then calculated the BSA between the two protein monomers (A and B, or a selection of the domains) using the following equation:

$$\mathrm{BSA}_{A\cap B} = \mathrm{SASA}_{A} - \mathrm{SASA}_{(A\cup B)\cap A}$$

Closer inspection revealed that the interaction between the two N-terminal β-barrels was mediated primarily by a group of polar and nonpolar residues in loops L1, L2, and L4 on the extracellular membrane leaflet (Fig. 3 *A*). The C-terminal periplasmic domains, on the other hand, formed a network of salt bridges involving residue K192 from one domain with residues E310 and E312 from the other domain (Fig. 3 *A*). Residue K192 could be cross-linked in vivo, whereas a K192A mutation abrogated the formation of

TABLE 2 BSA at the OmpA Dimerization Interface

	BSA (nm^2)			
	NTD	Linker	CTD	Total
Starting Model	11.58	0.00	5.84	17.42
OmpA	25.75 (1.00)	5.64 (0.45)	16.11 (1.01)	47.50 (1.49)
	25.89 (1.04)	0.82 (0.44)	16.48 (1.04)	43.19 (1.54)
OmpA^{1M}	28.05 (1.16)	3.79 (0.36)	22.76 (1.38)	54.60 (1.84)
	23.14 (1.00)	4.31 (0.69)	12.98 (1.05)	40.43 (1.61)

Values are given for the interaction surfaces between individual domains and the total BSA for each simulation replica (total for both monomers). Standard deviations are in parentheses.

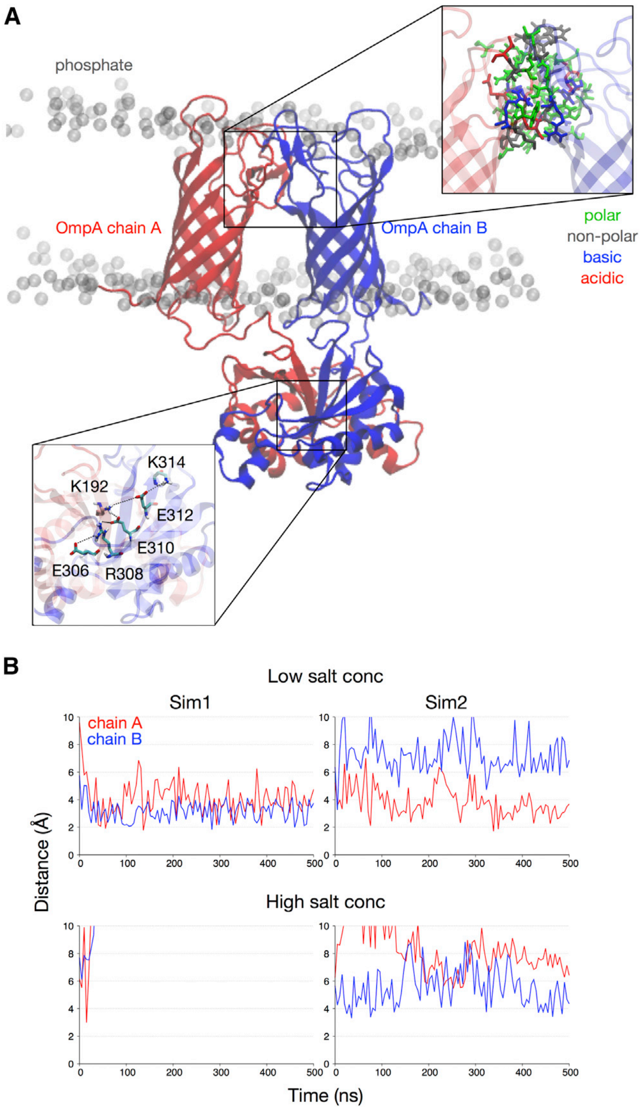

FIGURE 3 OmpA dimerization interface. (*A*) The N-terminal β-barrels dimerize via nonspecific polar and nonpolar interactions between residues found in the loop regions (*top inset*), whereas the C-terminal periplasmic domains interact via a network of salt bridges (*bottom inset*). (*B*) Distance between the side chains of two residues, K192 and E310, which are key to the formation of the dimerization interface in the CTDs. The colors represent the two monomers in (*A*). In one high-salt concentration simulation (*bottom left*), the distance between the residues was >10 Å after ~40 ns of simulation. To see this figure in color, go online.

dimers; therefore, our results provide further support for the notion that this residue is key for dimerization.

Although the K192-E310 salt bridge persisted throughout the entire simulation for both repeats of the system with lower ionic strength, increasing the salt concentration to 1 M abolished this interaction (Fig. 3 *B*). In the presence of an elevated ionic strength, the charges on the side chains of these lysine and glutamate residues were shielded by the Mg^{2+} and Cl^{-} ions, reducing the electrostatic attraction between them. To compensate for the loss of the salt-bridge network, the dimerization interface was stabilized via nonspecific polar interactions involving residues such as Q190, Q223, and N288. Carpenter et al. (33) observed a similar screening effect at a high salt concentration with OmpA from *P. multocida*: the CTD had fewer electrostatic interactions with the lipid headgroups compared with a similar system at a low ionic strength.

Membrane localization and lipid-protein interactions

A number of structural and computational studies of OmpA have suggested that the two aromatic rings on both the extracellular and periplasmic sides of the N-terminal β-barrel may mediate interactions with the local environment in the region where solvent, lipid headgroups, and hydrophobic tails coincide (1,41,42).

In our LPS-phospholipid bilayer model of the OM, the density of the aromatic belts on the periplasmic side was indeed located at the PG/PE lipid/water interface at both ionic strengths tested (Fig. 4 *A*). Residues Y129 and D90 in strand 6 and turn T2, respectively, sat face-on at this interface, keeping the barrels together (Fig. 4 *C*). On the extracellular side, the aromatic belt was located at the edge of the LPS headgroup/solvent interface, lying closer to the LPS tails (Fig. 4, *A* and *B*), and involved residues Y63, F15, and Y141, which helped to stabilize the dimerization interface. There was some limited water permeation into the interface between the loops and barrels on the extracellular side; at any given time, two to three water molecules penetrated into this region in the system with lower ionic strength, whereas ~10 molecules were found in the same

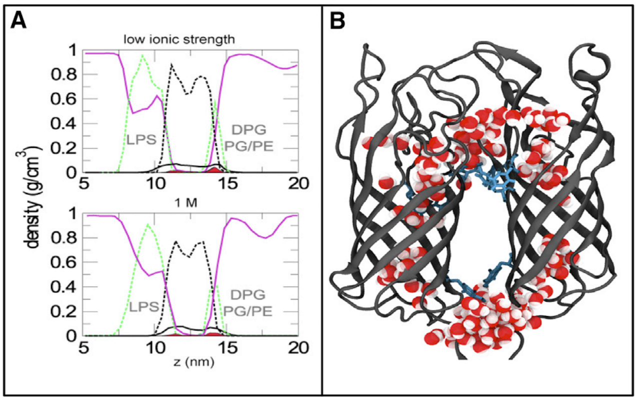

FIGURE 4 Stabilization of water-membrane interfaces by an aromatic belt. The trajectory-averaged density as a function of the *z* coordinate for the OmpA systems is shown. (*A*) The protein aromatic belts (*red solid areas*) can be seen to localize at the interface between the membrane lipid tails (*black dashed line*), the membrane headgroups (*green dashed line*), and water (*solid magenta line*). The overall N-terminal barrel protein density (*solid black line*) spans the whole membrane. (*B*) Close-up of the transmembrane domain, where the aromatic belt (with selected residues shown in *stick representation*) can be seen to lie on the interface of the periplasmic leaflet. On the extracellular side, water molecules permeate and aid the dimerization interface. To see this figure in color, go online.

area within the system with higher ionic strength. The close proximity of loops L2 and L4 led to a static funnel that encompassed water molecules around both pores (Fig. 4, *B* and *C*). The chains of the charged residues in these loops, D158 and R156, pointed toward the LPS headgroups, whereas the remainder of these two loops interacted primarily with the lipid tails of the LPS molecules.

The major difference between the systems at the two different ionic strengths was the salt distribution on the extracellular side of the membrane. From plots of the density of various molecular species as a function of the z coordinate (membrane normal; Fig. 4 *A*), it can be observed that in the 1 M cases the solvent penetrates farther into the bilayer, surrounding the entire belt of aromatic residues. In contrast, under low-salt conditions, only half of the aromatic belt is exposed to solvent, and there are two distinct areas of Mg^{2+} ions clustered around the phosphate groups of the heptose and glucosamine sugars of the inner core of the LPS. This is in agreement with previous simulation studies of LPS membranes (5,8).

A visual analysis of the transmembrane NTD indicated that lipid tails surrounded the majority of the N-terminal surface, with two tails sandwiched between the two barrels at the center of the membrane. On the periplasmic side of the barrel, the polar residues K3, D4, N5, Y43, D90, Y129, and T88 formed contacts with phospholipid headgroups. On the extracellular side, loops $L1^A$-$L1^B$ were buried in protein-protein interactions between chains (Fig. 4 *C*), contributing to the dimerization interface. Loops L2 and L3 formed a number of hydrogen bonds and salt bridges with the LPS sugars, whereas contacts between the LPS headgroup and loop L4 were few and far between (Fig. 5 *A*). The polar residues on loop L2 interacted with the alcohol, carboxyl, amide, and phosphate groups of the glucosamine moieties of LPS (Fig. 5 *B*). In contrast, loop L3 had the least solvent exposure, and formed contacts with the phosphate and alcohol groups of the heptose sugars of LPS instead (Fig. 5 *B*).

The structural integrity of the OM was maintained during all of the simulations. The importance of this observation is that in our simulations, despite the absence of peptidoglycan bound to the CTD, the full-length protein remained anchored within the membrane via the NTD, and did not perturb the membrane. The area per LPS acyl tail on the outer leaflet was 0.256 nm^2 and 0.273 nm^2 in low and 1 M salt solutions, respectively (Table 3), accounting for the cross-sectional area occupied by the two OmpA barrels (~16 nm^2) in the starting model. The higher value obtained in the 1 M solution may be a consequence of counterions penetrating deeper into the OM (cf. Fig. 4) and causing the LPS sugars to be cross-linked by multiple counterions and hence farther away from each other. However, experimental testing would be needed to clarify this. Overall, an area per acyl tails of 0.256 nm^2 is agreement with previous experimental estimates of 0.260 nm^2 for the area per lipid tail of LPS (43), and simulations of the OM of *E. coli*, which yielded average values of 0.251 nm^2 (20).

The periplasmic leaflet contained a 135:8:7 mixture of PE, PG, and DPG lipids, respectively. Consequently, the area per lipid acyl tail was calculated. The mean area per phospholipid acyl tail in the inner leaflet was 0.303 nm^2, which is in line with previous studies. Two modeling studies reported the area per lipid tail of DMPG and POPG in simulations to be 0.283 nm^2 and 0.301 nm^2, respectively, using the CHARMM27 (11) and GROMOS force fields (20). Additionally, experimental studies have reported values of ~0.303 nm^2 for DPPE (44), and 0.305 nm^2 at 313 K (45) and 0.283 nm^2 at 303 K (46) for POPE.

The bilayer thickness was ~3.7 nm, as measured from the phosphate groups on the periplasmic leaflet to the lipid A

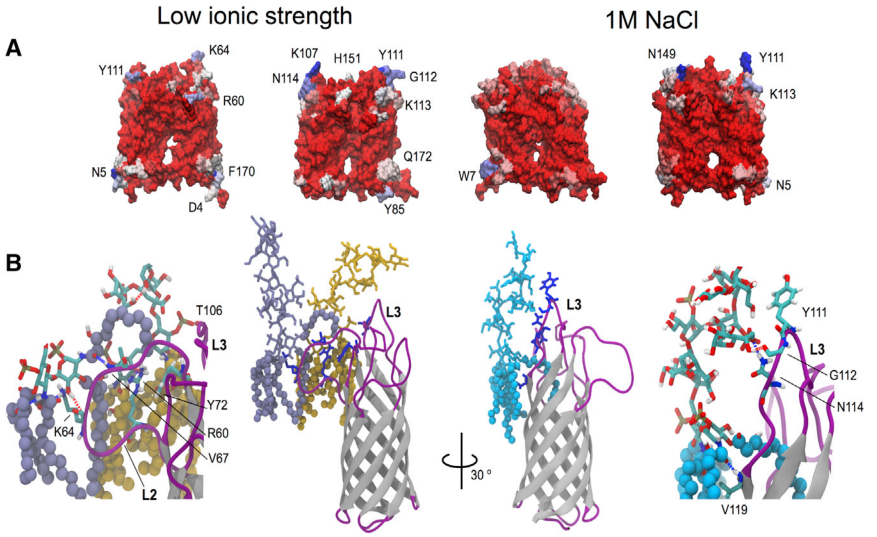

FIGURE 5 Protein interactions with LPS. (*A*) The surface of the protein is colored according to the number of contacts (where each contact has an interatomic distance of ≤0.35 nm) between the OmpA barrel residues and LPS headgroup atoms. The numbers of contacts are represented on an RWB scale, where red indicates zero contacts with the lipid groups and dark blue indicates the residue that makes contacts in 100% of the frames. Residues with contact frequencies of >50% are labeled. (*B*) Interaction of LPS with the extracellular loops of OmpA. The relative position of OmpA is shown next to the sugar LPS groups. To see this figure in color, go online.

TABLE 3 Membrane Properties

	Area per Acyl Tail (nm^2)		
	LPS	PE/PG/DPG	Membrane Thickness (nm)
OmpA	0.256 (0.003)	0.303 (0.001)	3.67 (0.01)
OmpA^{1M}	0.273 (0.003)	0.323 (0.001)	3.76 (0.02)

The area per lipid and the membrane thickness were calculated for the last 50 ns of the simulation and averaged over two independent simulations. The area per lipid was calculated for both leaflets, constituting 59 LPS molecules on the outer leaflet, and 135 PG, 8 PE, and 7 DPG molecules on the inner leaflet. Errors are shown in parentheses.

phosphate groups of LPS on the outer leaflet. Simulations in 1 M salt showed similar protein-membrane behavior, with the addition of decreased loop mobility due to the counterion contacts close to the highly charged LPS sugar headgroups. Compared with the low-ionic-strength simulations, both the area per acyl tail and the membrane thickness increased by ~0.02 nm^2 and ~0.1 nm, respectively. This corroborates previous experimental findings that reconstituted phospholipid bilayers swelled in a salt solution, and that this was driven primarily by weakening of the van der Waals attraction, without altering the membrane structure or bending rigidity (15).

As mentioned above with regard to the conformational dynamics analysis, in one simulation of the system in the presence of only neutralizing counterions, substantial interactions of the CTDs with the periplasmic leaflet of the membrane were observed. These interactions were a combination of nonspecific polar interactions and salt bridges. The salt bridges involved the phosphate groups of PE lipids and residues K267 and K294, and the choline group of a PE lipid and residue D291. Once the interactions formed (after ~300 ns for D291 and K267, and after ~400 ns for K294), they were stable for the remainder of the simulation. The time-dependent distances between the key groups in these interactions are shown in Fig. 6.

Pore gating

Studies of OmpA, both in vitro and in silico, have shown that the β-barrel can be in either an open or a closed state, giving rise to different levels of conductance. The transition between these states is due to the dynamics of the key salt bridges R138-E52 and E128-K82 (1,8). In an experimental work, Hong et al. (3) measured the interaction energies between these side chains via a double-mutant analysis, and concluded that the opening mechanism was caused by the K82 side chain moving up to E52 and destabilizing the R138-E52 bridge to form a bridge between K82 and E52 instead. Regardless of the mechanism, both our study and that of Hong et al. indicate that R138-E52 is the gating salt bridge in OmpA. Although the model we used in this work (taken from Marcoux et al. (11)) started in the open state, with a preformed E52-K82 salt bridge, a R138-E52

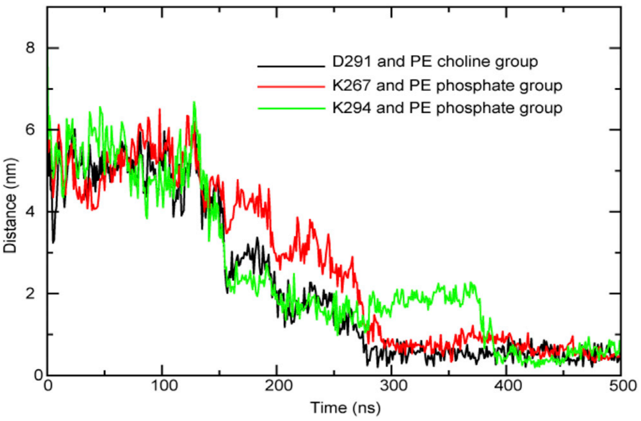

FIGURE 6 Interactions of the CTD with the periplasmic leaflet of the membrane. The distances between the choline or phosphate moiety of PE lipids and three charged residues are a shown as a function of time. Once formed, all three interactions were stable for >100 ns. To see this figure in color, go online.

bridge formed quickly. This bridge remained the most prevalent interaction, interrupted by a couple of pore-opening events as R138 interacted with E128 (Fig. 7 *A*). Both R138-E52 salt bridges were long-lived when only neutralizing ions were present, with a water molecule passing through the pores every few nanoseconds (Fig. 7 *B*).

In the second independent simulation, a pore-opening event was observed at 420 ns in which the salt bridge was disrupted, leading to a sudden influx of ~70 water molecules. This system had a total flux of 270 water molecules, whereas the first replica resulted in a total of ~210 molecules in 500 ns. This indicated an interplay between meta-stable open and closed states, as previously suspected (3). The simulations performed in 1 M salt had even fewer molecules moving across the membrane (~100; Fig. 7 *A*), despite some temporary disruption of the R138-E52 bridge. We did not observe the passage of ions through the entire pore in any of the simulations.

We note that in the simulations of the full-length monomer, we observed a similar pore-gating mechanism, i.e., the opening of the R138-E52 bridge resulted in an influx of water molecules (Fig. S3). The pore-opening events in these simulations, however, were also facilitated by the interaction between E52 and K82 residues, apart from the R138-E128 salt bridge previously observed in the dimer simulations, suggesting a stochastic nature of salt-bridge formations within the pore to control the flow of water. Despite this difference, the total cumulative flux in the monomer simulations was comparable to that observed in the dimer simulations, with ~50 water molecules passing through after 100 ns.

CONCLUSIONS

In this study, we investigated the stability of a model of full-length OmpA in an asymmetric LPS/phospholipid

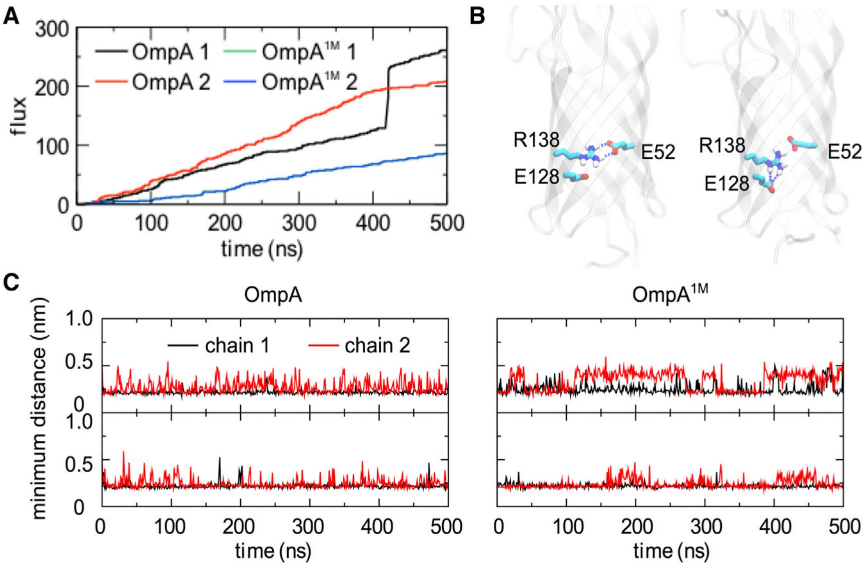

FIGURE 7 Water transport through the transmembrane OmpA NTD. (*A*) Total flux of water molecules through the NTD barrels as a function of the simulation time. (The *blue and green curves* are essentially identical and thus only the *blue* one is visible). A similar analysis performed for simulations of monomeric OmpA is provided in Fig. S3. (*B*) OmpA NTD showing the key residues involved in the gating of the pore in both closed (*left*) and open (*right*) configurations. (*C*) Minimum distance between the pore-gating residue pair R138 and E52 for all simulations. To see this figure in color, go online.

membrane. The low RMSD and RMSF values observed in the monomer simulations indicate that the model itself is structurally robust, and the large BSA found between the two monomers in the dimer simulations suggests a stable dimerization state. Dimerization is mediated by nonspecific polar and hydrophobic interactions between the NTDs and a network of salt bridges between the CTDs. The interaction of the loops of the two monomers with each other, on the extracellular side of the membrane, mediated a large proportion of the protein-protein interactions, whereas the turns on the periplasmic side interacted with the phospholipid headgroups as well as the linker region of OmpA. Although the NTDs and CTDs showed little structural deviation, the linker was flexible, stretching or contracting to a number of different lengths as the CTDs remained in solvent or approached the membrane.

In agreement with previous simulation studies of the transmembrane domain of OmpA and indeed other OMPs, the aromatic residues that formed a belt around the β-barrels remained at the interface between the solvent and the LPS/phospholipid membrane. However, on the extracellular side, the aromatic belt also mediated protein-lipid interactions and encompassed a region of water around the dimerization interface, away from LPS molecules. This has not previously been reported in the literature.

The interaction of the loops on the extracellular side of the OM resulted in the L1 loops being buried together, L4 loops interacting with the LPS tails, L2 interacting with the tails and glucosamine headgroup, and L3 interacting with the heptose sugars only. To the best of our knowledge, these are the first reported insights into the LPS interactions of the OmpA NTD.

We also studied the role of OmpA as a porin, particularly with regard to what effects dimerization and inclusion of the full-length protein had on water transport through the membrane. Disruption of the R138-E52 salt bridge, located on the inside of the N-terminal barrel, led to the formation of an alternative salt bridge to compensate for the loss of favorable electrostatics. In our simulations of the dimeric OmpA systems, the alternative salt bridge was R138-E128. This is in contrast to previous experimental and simulation studies by Hong et al. (3) and Khalid et al. (4), respectively, which instead showed the formation of an E52-K85 salt bridge. This difference may be a consequence of the more realistic OM environment we used in this study, or an effect of dimerization. Of course, it is perfectly feasible that either one of the alternative salt bridges may form, as demonstrated by Bond et al. (1) and in our simulations of the full-length monomeric OmpA. The total flux through the membrane was ~210–260 molecules over 500 ns, although the full disruption of the bridge during an ~1 ns period showed a spike in the flux of 70 molecules.

In general, the conformational behavior of the dimers was similar at both of the ionic strengths we studied. However, there were some differences, mainly in the overall deviations from the starting model and the per-residue fluctuations, both of which were lower in 1 M salt due to the greater number of counterions. This also resulted in ~50 fewer water molecules permeating through the protein. The counterions also shielded the CTDs from the membrane, resulting in smaller movements of the linker region.

Thus, this study provides insight into the dimerization interface of the full-length OmpA, and opens up possibilities for studying the *E. coli* cell envelope, which is crucial for designing novel antibiotics. Importantly, the simulations described here provide questions to guide further experimental studies. Can the formation of dimers be prevented by mutation of residues within the dimerization interfaces of the NTDs and CTDs? Does the mutation of residues in one of the domains prevent dimerization or do both domains

need to be mutated? The challenge then will be to link these questions to the functional role of the dimer, and thus this protein remains an enigmatic subject of investigation.

SUPPORTING MATERIAL

Three figures are available at http://www.biophysj.org/biophysj/supplemental/S0006-3495(16)30802-5.

AUTHOR CONTRIBUTIONS

M.L.O.-S., F.S., and T.J.P. performed the research. P.J.B. and S.K. designed the research. M.L.O.-S., F.S., and S.K. analyzed the data. M.L.O.-S., F.S., P.J.B., and S.K. wrote the manuscript.

ACKNOWLEDGMENTS

We thank Julien Marcoux and Carol Robinson for providing the coordinates of their OmpA dimer and monomer models.

This work was supported by a grant from the Life Sciences Interface, University of Southampton, and made use of the IRIDIS High Performance Computing Facility at the University of Southampton.

REFERENCES

1. Bond, P. J., J. Faraldo-Goméz, and M. S. P. Sansom. 2002. OmpA: a pore or not a pore? Simulation and modelling studies. *Biophys. J.* 83:763–775.
2. Fernandez, C., C. Hilty, …, K. Wuthrich. 2001. Solution NMR studies of the integral membrane proteins OmpX and OmpA from *Escherichia coli*. *FEBS Lett.* 504:173–178.
3. Hong, H., A. Szabo, and L. K. Tamm. 2006. Electrostatic couplings in OmpA ion-channel gating suggest a mechanism for pore opening. *Nat. Chem. Biol.* 2:627–635.
4. Khalid, S., P. J. Bond, …, M. S. Sansom. 2008. OmpA: gating and dynamics via molecular dynamics simulations. *Biochim. Biophys. Acta.* 1778:1871–1880.
5. Pautsch, A., and G. E. Schulz. 2000. High-resolution structure of the OmpA membrane domain. *J. Mol. Biol.* 298:273–282.
6. Ramakrishnan, M., J. Qu, …, D. Marsh. 2005. Orientation of beta-barrel proteins OmpA and FhuA in lipid membranes. Chain length dependence from infrared dichroism. *Biochemistry.* 44:3515–3523.
7. Sušac, L., R. Horst, and K. Wuthrich. 2014. Solution-NMR characterization of outer- membrane protein A from E. coli in lipid bilayer nanodiscs and detergent micelles. *ChemBioChem.* 15:995–1000.
8. Arora, A., F. Abildgaard, …, L. K. Tamm. 2001. Structure of outer membrane protein A transmembrane domain by NMR spectroscopy. *Nat. Struct. Biol.* 8:334–338.
9. Grizot, S., and S. K. Buchanan. 2004. Structure of the OmpA-like domain of RmpM from *Neisseria meningitidis*. *Mol. Microbiol.* 51:1027–1037.
10. Park, J. S., W. C. Lee, …, H. Y. Kim. 2012. Mechanism of anchoring of OmpA protein to the cell wall peptidoglycan of the gram-negative bacterial outer membrane. *FASEB J.* 26:219–228.
11. Marcoux, J., A. Politis, …, C. V. Robinson. 2014. Mass spectrometry defines the C-terminal dimerization domain and enables modeling of the structure of full-length OmpA. *Structure.* 22:781–790.
12. Kaspersen, J. D., C. M. Jessen, …, J. S. Pedersen. 2014. Low-resolution structures of OmpA.DDM protein-detergent complexes. *ChemBioChem.* 15:2113–2124.
13. Wang, Y. 2002. The function of OmpA in *Escherichia coli*. *Biochem. Biophys. Res. Commun.* 292:396–401.
14. Torres, A. G., and J. B. Kaper. 2003. Multiple elements controlling adherence of enterohemorrhagic Escherichia coli O157:H7 to HeLa cells. *Infect. Immun.* 71:4985–4995.
15. Weiser, J. N., and E. C. Gotschlich. 1991. Outer-membrane protein A (OmpA) contributes to serum resistance and pathogenicity of Escherichia coli K-1. *Infect. Immun.* 59:2252–2258.
16. Raetz, C. R. H. 1990. Biochemistry of endotoxins. *Annu. Rev. Biochem.* 59:129–170.
17. Vinogradov, E. V., K. Van Der Drift, …, O. Holst. 1999. The structures of the carbohydrate backbones of the lipopolysaccharides from Escherichia coli rough mutants F470 (R1 core type) and F576 (R2 core type). *Eur. J. Biochem.* 261:629–639.
18. Appelmelk, B. J., Y. Q. An, …, J. de Graaf. 1994. Frequencies of lipopolysaccharide core types in Escherichia coli strains from bacteraemic patients. *Microbiology.* 140:1119–1124.
19. Lugtenberg, E. J., and R. Peters. 1976. Distribution of lipids in cytoplasmic and outer membranes of Escherichia coli K12. *Biochim. Biophys. Acta.* 441:38–47.
20. Piggot, T. J., D. A. Holdbrook, and S. Khalid. 2011. Electroporation of the E. coli and S. Aureus membranes: molecular dynamics simulations of complex bacterial membranes. *J. Phys. Chem. B.* 115:13381–13388.
21. Pronk, S., S. Pall, …, E. Lindahl. 2013. GROMACS 4.5: a high-throughput and highly parallel open source molecular simulation toolkit. *Bioinformatics.* 29:845–854.
22. Bekker, H., H. J. C. Berendsen, …, M. K. R. Renardus. 1993. Gromacs—a parallel computer for molecular-dynamics simulations. *In* Physics Computing ’92. R. A. DeGroot and J. Nadrchal, editors. World Scientific Publishing, Singapore, pp. 252–256.
23. Oostenbrink, C., T. A. Soares, …, W. F. van Gunsteren. 2005. Validation of the 53A6 GROMOS force field. *Eur. Biophys. J.* 34:273–284.
24. Berweger, C. D., W. F. van Gunsteren, and F. Mullerplathe. 1995. Force-field parametrization by weak coupling—reengineering SPC water. *Chem. Phys. Lett.* 232:429–436.
25. Nose, S., and M. L. Klein. 1983. Constant pressure molecular-dynamics for molecular systems. *Mol. Phys.* 50:1055–1076.
26. Hoover, W. G. 1985. Canonical dynamics: equilibrium phase-space distributions. *Phys. Rev. A.* 31:1695–1697.
27. Nose, S. 1984. A unified formulation of the constant temperature molecular-dynamics methods. *J. Chem. Phys.* 81:511–519.
28. Piggot, T. J., D. A. Holdbrook, and S. Khalid. 2013. Conformational dynamics and membrane interactions of the E. coli outer membrane protein FecA: a molecular dynamics simulation study. *Biochim. Biophys. Acta.* 1828:284–293.
29. Parrinello, M., and A. Rahman. 1981. Polymorphic transitions in single crystals—a new molecular dynamics method. *J. Appl. Phys.* 52:7182–7190.
30. Essmann, U., L. Perera, …, L. G. Pedersen. 1995. A smooth particle mesh Ewald method. *J. Chem. Phys.* 103:8577–8593.
31. Hess, B., H. Bekker, …, J. G. E. M. Fraaije. 1997. LINCS: a linear constraint solver for molecular simulations. *J. Comput. Chem.* 18:1463–1472.
32. Kukol, A. 2009. Lipid models for united-atom molecular dynamics simulations of proteins. *J. Chem. Theory Comput.* 5:615–626.
33. Carpenter, T., S. Khalid, and M. S. Sansom. 2007. A multidomain outer membrane protein from Pasteurella multocida: modelling and simulation studies of PmOmpA. *Biochim. Biophys. Acta.* 1768:2831–2840.
34. Hwang, P. M., W. Y. Choy, …, L. E. Kay. 2002. Solution structure and dynamics of the outer membrane enzyme PagP by NMR. *Proc. Natl. Acad. Sci. USA.* 99:13560–13565.

35. Ahn, V. E., E. I. Lo, …, G. G. Prive. 2004. A hydrocarbon ruler measures palmitate in the enzymatic acylation of endotoxin. *EMBO J.* 23:2931–2941.

36. Vandeputte-Rutten, L., M. P. Bos, …, P. Gros. 2003. Crystal structure of Neisserial surface protein A (NspA), a conserved outer membrane protein with vaccine potential. *J. Biol. Chem.* 278:24825–24830.

37. Snijder, H. J., R. L. Kingma, …, B. W. Dijkstra. 2001. Structural investigations of calcium binding and its role in activity and activation of outer membrane phospholipase A from *Escherichia coli*. *J. Mol. Biol.* 309:477–489.

38. Bockmann, R. A., and A. Caflisch. 2005. Spontaneous formation of detergent micelles around the outer membrane protein OmpX. *Biophys. J.* 88:3191–3204.

39. Cox, K., and M. S. Sansom. 2009. One membrane protein, two structures and six environments: a comparative molecular dynamics simulation study of the bacterial outer membrane protein PagP. *Mol. Membr. Biol.* 26:205–214.

40. Cox, K., P. J. Bond, …, M. S. Sansom. 2008. Outer membrane proteins: comparing X-ray and NMR structures by MD simulations in lipid bilayers. *Eur. Biophys. J.* 37:131–141.

41. Ulmschneider, M. B., and M. S. P. Sansom. 2001. Amino acid distributions in integral membrane protein structures. *Biochim. Biophys. Acta.* 1512:1–14.

42. Yau, W. M., W. C. Wimley, …, S. H. White. 1998. The preference of tryptophan for membrane interfaces. *Biochemistry.* 37:14713–14718.

43. Snyder, S., D. Kim, and T. J. McIntosh. 1999. Lipopolysaccharide bilayer structure: Effect of chemotype, core Mutations, divalent cations, and temperature. *Biochemistry.* 38:10758–10767.

44. Petrache, H. I., S. W. Dodd, and M. F. Brown. 2000. Area per lipid and acyl length distributions in fluid phosphatidylcholines determined by 2H NMR spectroscopy. *Biophys.* 79:3172–3192.

45. Rappolt, M., A. Hickel, …, K. Lohner. 2003. Mechanism of the lamellar/inverse hexagonal phase transition examined by high resolution x-ray diffraction. *Biophys.* 84:3111–3122.

46. Rand, R. P., N. Fuller, …, D. C. Rau. 1988. Variation in hydration forces between neutral phospholipid bilayers: evidence for hydration attraction. *Biophys.* 27:7711–7722.

47. Daura, X., K. Gademann, …, A. E. Mark. 1988. Peptide folding: when simulation meets experiment. *Angew. Chem. Int. Ed.* 1999:236–240.

Biophysical Journal, Volume *111*

Supplemental Information

Full-Length OmpA: Structure, Function, and Membrane Interactions Predicted by Molecular Dynamics Simulations

Maite L. Ortiz-Suarez, Firdaus Samsudin, Thomas J. Piggot, Peter J. Bond, and Syma Khalid

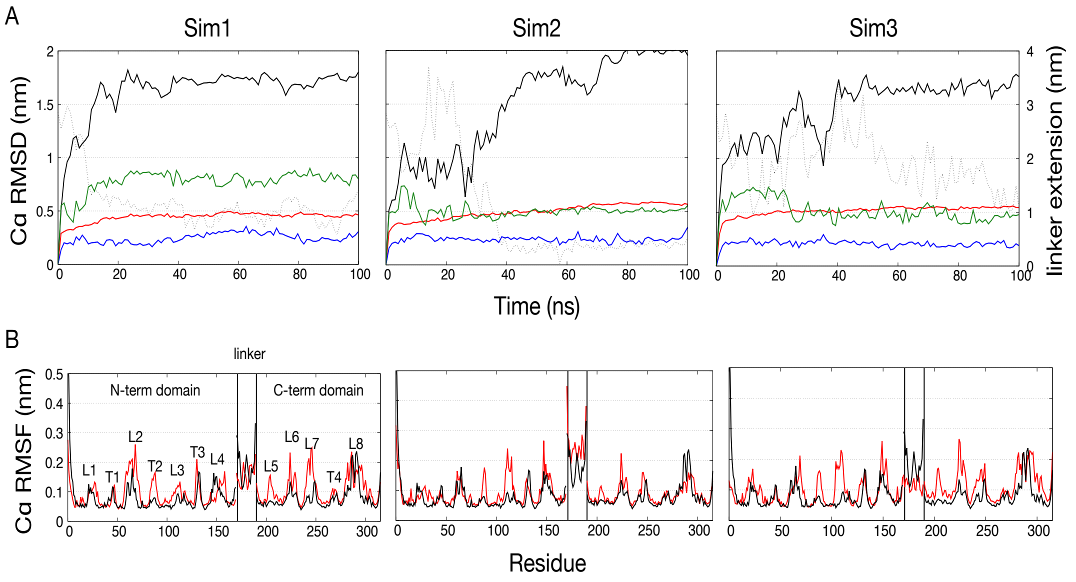

Supp. Figure 1: OmpA monomer structural stability. (A) Cα RMSD of the full length OmpA monomer (black) and its domains, i.e. N-terminal domain (red), linker (green) and C-terminal domain (blue). The extension of the linker region is depicted as black dashed line. (B) Per-residue Cα RMSF of OmpA monomer from the isolated monomeric simulation (red), compared to that from dimeric simulation (black). Each domain is separated by the black vertical lines.

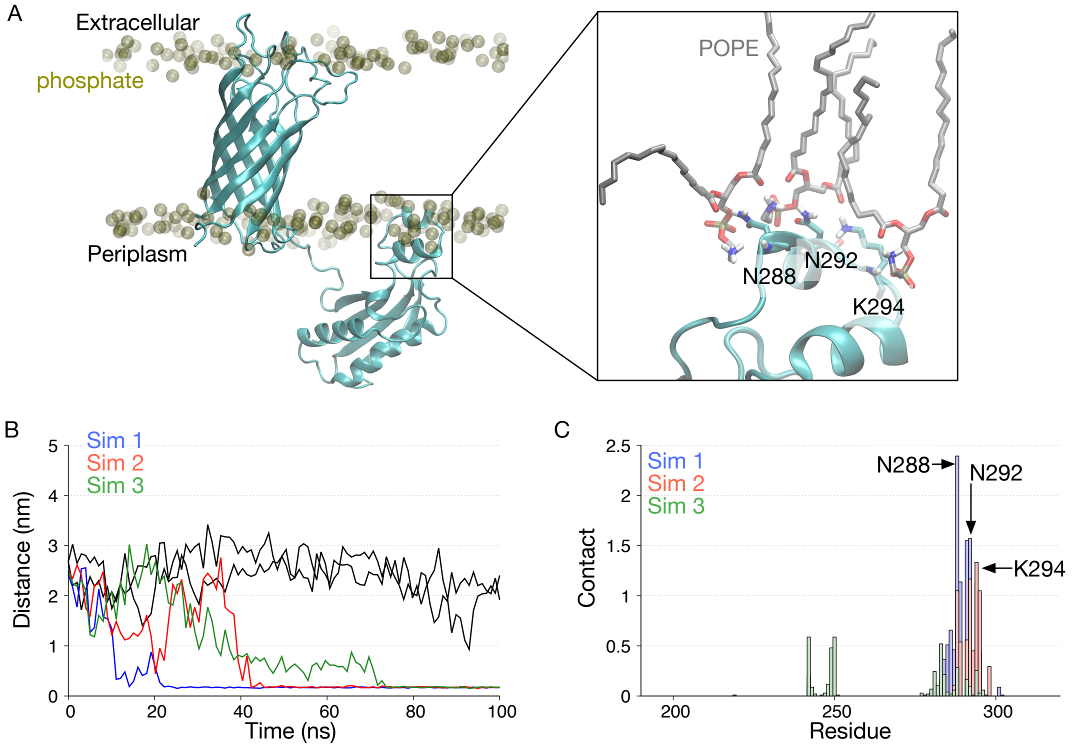

Supp. Figure 2: OmpA C-terminal domain interacts with the membrane. (A) Representative structure of OmpA monomer with respect to the membrane (phosphate atoms shown as brown spheres) at the end of the 100 ns simulations. Enlarged figure highlights key residues involved in the interactions. (B) Minimum distance between the C-terminal domain (residue 188-316) and the inner leaflet of the outer membrane throughout the 100 ns simulations (blue, red and green), compared to that of the dimer simulations (black). (C) Contact analysis performed for each residue of the C-terminal domain, presented as the average number of lipids found within 3.5 Å of the residue.

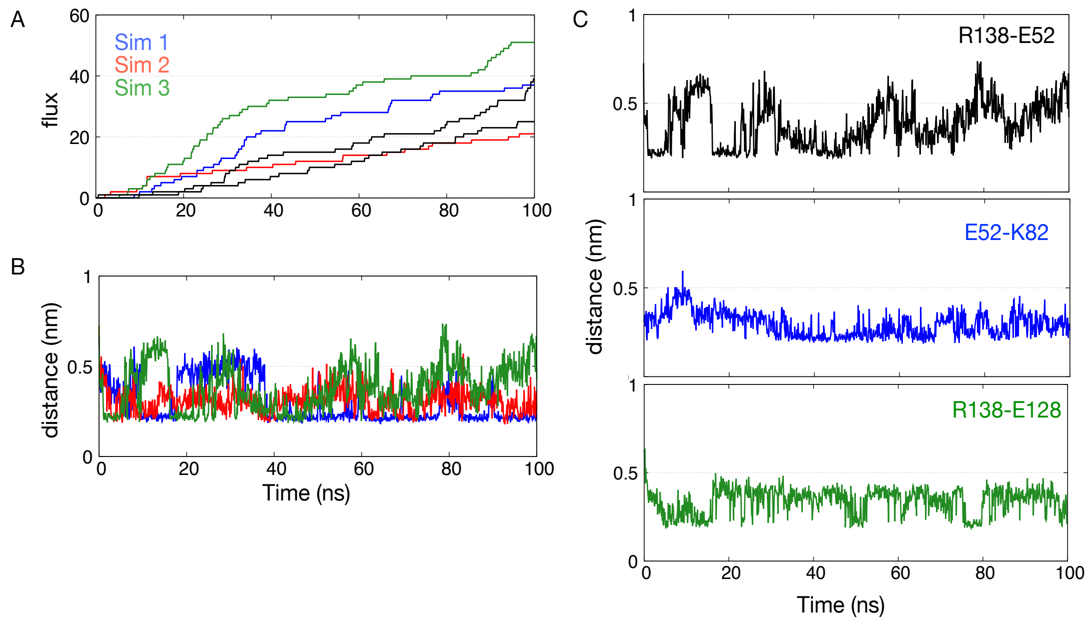

Supp. Figure 3: Water transport through the monomeric OmpA. (A) Total flux of water molecules through the N-terminal β-barrel throughout the 100 ns simulation of OmpA monomer (blue, red and green lines), compared to that of OmpA dimer (black lines). (B) The corresponding opening and closing of the R138-E52 gate depicted as the minimum distance between these residues. (C) The interactions between these pore gating residues with two other key residues in Simulation 3, shown as minimum distance between E52 and K82 (blue), and R138 and E128 (green).
